# Role of Non-Steroidal Anti-Inflammatory Drugs in Gynecology

**DOI:** 10.3390/ph3072082

**Published:** 2010-07-05

**Authors:** Anna Livshits, Daniel S. Seidman

**Affiliations:** 1 Department of Obstetrics and Gynecology, Medical College of Wisconsin, Milwaukee, WI, USA; E-Mail:alivshits@mcw.edu (A.L.); 2 Department of Obstetrics and Gynecology, the Chaim Sheba Medical Center, and the Sackler School of Medicine, Tel Aviv University, Tel Aviv, Israel

**Keywords:** non-steroidal anti-inflammatory drugs, dysmenorrhea, menorrhagia

## Abstract

This review summarizes the current use of non-steroidal anti-inflammatory drugs (NSAIDs) in obstetrics, gynecology and infertility. These medications are commonly used in different fields of reproductive medicine, for pain management after operative procedures and to relieve dysmenorrhea. In addition to their analgesic effect, NSAIDs are helpful in the management of menorrhagia by decreasing menstrual blood loss. NSAIDs alleviate pain associated with medical abortion, assist in undertaking natural cycle *in-vitro* fertilization by preventing follicular rupture and reducing premature ovulation, and serve as tocolytics in preterm labor. New NSAIDs may have a growing role in management of women's health.

## 1. Introduction

Non-steroidal anti-inflammatory drugs (NSAIDs) are commonly used first line drugs for treatment of different medical situations in the field of obstetrics and gynecology. Their anti-inflammatory properties make them most useful in the management of disorders in which pain is related to the intensity of the inflammatory process. Most currently available traditional NSAIDs act by inhibiting the prostaglandin G/H synthase enzymes, known as the cyclooxygenases. Those enzymes convert arachidonic acid to the unstable intermediates prostaglandin G_2_ and prostaglandin H_2_ and lead to the production of thromboxane A_2_ and a variety of other prostaglandins which contribute to pain. At higher concentrations, NSAIDs are also known to reduce production of superoxide radicals, induce apoptosis, inhibit the expression of adhesion molecules, decrease nitric oxide synthase, decrease proinflammatory cytokines (e.g., TNF-, interleukin-1), modify lymphocyte activity and alter cellular membrane functions. In obstetrics and gynecology, NSAID’s have long been used to control acute and chronic postoperative pain, menstrual pain, pain related to medical abortions, menorrhagia, intrauterine device, assist in fertility treatment, and administered as tocolytics in preterm labor. The benefits of NSAIDs for specific gynecological diagnoses and the type of methodology used to establish efficacy are summarized in Table 1.

**Table 1:** Benefits of NSAIDs for specific gynecological diagnoses and the type of methodology used to establish efficacy.

Medical Implementation	Efficacy	Methodology & References
Primary Dysmenorrhea	Highly efficient, up to 85% in pain reduction	RCT* [1,2,3,6]
Menorrhagia	30-40% in bleeding reduction	RCT [ 7,8,9,10]
Pain/heavy bleeding associated with intrauterine device (IUD)	Up to 82% pain reduction, up to 57% in bleeding reduction	RCT [12]
Pain associated with medical abortion	Up to 48% in pain reduction	RCT [ 13,14,15]
Infertility	10% reduction in premature ovulation	Retrospective study [18,19,20]
Tocolysis	Significant reduction in preterm deliveries. Relative Risk 0.21.	RCT [ 27,28]

*RCT=Randomized controlled ­studies.

## 2. Primary Dysmenorrhea

Primary dysmenorrhea is cyclic menstrual pain, usually described as “cramping”, without an identifiable associated pathology. Primary dysmenorrhea more commonly begins within two years after menarche, often accompanied by low backache, nausea and vomiting, headache, or diarrhea. Research has shown that women with dysmenorrhoea have high levels of prostaglandins, hormones known to cause cramping abdominal pain [1]. The most common treatment for primary dysmenorrhea are NSAIDs. They decrease menstrual pain by decreasing intrauterine pressure and lowering prostaglandin F2 alpha levels in menstrual fluid. NSAIDs relief can be obtained in 80-85% of patients studied [2]. Two meta-analyses [3,4] of randomized controlled trials (RCTs) of NSAIDs *versus* acetaminophen found that all of the NSAIDs studied (*i.e.*, ibuprofen, naproxen, mefenamic acid, and aspirin) were effective in treating women with dysmenorrhea, and all of the NSAIDs were more effective than acetaminophen [5]. A Cochrane database review between 1966 and 2003 assessed RCTs of NSAID therapies *versus* placebo for primary dysmenorrhea and found that NSAIDs were significantly more effective for pain relief than placebo (OR 7.91, 95% CI 5.65 to 11.09), with little evidence of the superiority between any individual NSAIDs [1]. Another analysis of five trials was performed to assess efficacy of NSAIDs compared to acetaminophen and placebo. It demonstrated that NSAID’s provided greater pain relief than acetaminophen and placebo within 30 minutes of administration (P < 0.01 and P < 0.05, respectively) [6].

## 3. Menorrhagia

Menorrhagia is defined as prolonged (more than 7 days) or heavy (more than 80 mL) cyclic menstruation. Excessive prostaglandin synthesis has also been implicated in primary menorrhagia [7]. The endometrium of women with excessive menstrual bleeding has been found to have higher levels of prostaglandin E2 and F2a when compared with women with normal menses. There is further evidence of deranged haemostasis (abnormal clotting) as the ratio of prostaglandin E2 to F2 and the ratio of prostacyclin (prostaglandin I2) to thromboxane are elevated [8]. These substances are present both in the endometrium and myometrium, although the exact mechanism by which the excessive blood loss occurs remains speculative. 

NSAIDs in adequate dosages decrease ovulatory bleeding by approximately 30-40% with less reduction in anovulatory cycles [9]. Cochrane review of nine RCTs conducted between 1996 and 2007 that were investigating effectiveness of NSAIDs in achieving a reduction in menstrual blood loss in women of reproductive years with menorrhagia, showed good results with the use of NSAIDs [10]. As a group, NSAIDs were more effective than placebo at reducing heavy menstrual bleeding but less effective than either tranexamicacid, danazol or the levonorgestrel releasing intrauterine system. Nevertheless, treatment with danazol caused more adverse events than NSAIDs. There were no statistically significant differences between NSAIDs and the other treatments (oral luteal progestogen, ethamsylate, an older progesterone releasing intra-uterine system (Progestasert oral contraceptive pill), but most studies were underpowered [10].

## 4. Pain or Heavy Bleeding Associated with Intrauterine Device

Intrauterine device (IUD) is the most common method of reversible contraception worldwide [11]. Pain and abnormal uterine bleeding are frequently encountered to be the reason for premature discontinuation of IUD. Cochrane systematic review of 2009 found that NSAIDs were effective in reducing menstrual blood loss and the pain associated with IUD use [12]. This review included 15 trials from 10 countries, using different NSAIDs (naproxen, suprofen, mefenamic acid, ibuprofen, indomethacin, flufenamic acid, alclofenac, and diclofenac). NSAIDs decreased the amount of menstrual blood loss not only in women with complains of menorrhagia, but also in women with regular bleeding with IUD. On the other hand, there were mixed results about prophylactic use of NSAIDs. Studies didn’t find effect on decreasing the pain and menstrual bleeding with NSAIDs administration at the time of IUD insertion [12]. 

## 5. Pain Associated with Medical Abortion

Performance of medical abortions with mifepristone, progesterone antagonist, or methotrexate, followed by administration of prostaglandin analogue misoprostol has gained popularity during the last two decades. One of the major side effects of medical abortion is abdominal pain in response to misoprostol. NSAIDs were often avoided to treat this side effect, because of concern over their potential inhibition of prostaglandin-induced uterine contractions. However, recent studies have not shown interference by ibuprofen on the action of methotrexate and misoprostol in medical abortions at up to 56 gestational days [13]. In another study NSAIDs controlled the pain caused by medical abortion induced by mifepristone and misoprostol, without attenuating the efficacy of the two drugs [14]. An additional study the investigated first trimester medical abortions with the same combination of mifepristone and misoprostol also supported the use of NSAIDs for pain control [15]. The study compared ibuprofen with paracetamol for pain relief, and found that NSAIDs were significantly more effective in reducing pain during medical abortion, without interfering with the action of misoprostol [15]. 

## 6. Infertility

The use of NSAIDs can induce reversible infertility in humans, underlining the observation that COX inhibition prevents normal reproductive processes. Young women are receiving NSAIDs for different indications such as inflammatory joint disease, developed luteinized unruptured ovarian follicles [16,17]. Normal ovulation returned after NSAIDs were withdrawn. This effect can paradoxically be helpful in planning and timing *in-vitro* fertilization (IVF) treatments. The use of indomethacin to reduce the incidence of spontaneous ovulation in natural IVF cycles have been supported by several studies [18,19]. Indomethacin was able to prevent follicular rupture and reduce premature ovulation, with an increase in oocyte retrieval rate. Later, prospective trial showed a lower spontaneous ovulation rate among 42 natural IVF cycles, who received human chorionic gonadotropins (HCG) and indomethacin (50 mg three times daily for three days), compared with139 cycles with HCG alone [20]. Indomethacin was used in those cycles that were predicted to ovulate during weekend, by follicle size ≥ 16 mm and those who had a spontaneous LH surge on weekend, when fertility clinic was unable to perform retrieval. Although the difference was not significant between the two groups, the patients who took indomethacin were able to delay oocyte retrieval for up to 72 h.

Another retrospective study evaluated the number of spontaneous ovulations occurring before oocyte retrieval in modified natural cycle IVF (using gonadotrophin releasing hormone antagonist, human menopausal gonadotrophin and HCG) with and without the use of indomethacin [19]. Indomethacin was administered for three days (50 mg three times a day) after the leading follicle reached 14 mm size. There were 84 cycles with indomethacin and 171 without indomethacin. The number of cycles with ovulation before oocyte retrieval and the number of cycles with no oocytes at retrieval were assessed with and without indomethacin. In addition, the pregnancy rates in the two groups of patients were analysed. Results from this study show that the use of indomethacin in modified natural IVF cycles reduces premature ovulation from 16% (without indomethacin) to 6%.

## 7. NSAIDs as Tocolytics

Prostaglandins play an important role in endometrial function. Pregnant human myometria express both cyclooxygenase-1 (COX-1) and cyclooxygenase-2 (COX-2) [21]. Throughout pregnancy, COX-1 expression is low in human amnion and chorion decidua and does not change with gestational age [22]. However, COX-2 expression in fetal membranes increases with gestational age, and increases significantly before, and during, labor. Increased COX-2 expression precedes the onset of labor, suggesting that it may be involved in the initiation of labor, and in the increase of prostaglandin synthesis within the fetal membranes at term [23]. The increase in prostaglandin during labor causes increased uterine contractility and inhibitors of prostaglandin synthesis delay the onset of labor [24,25,26].

NSAIDs have proven to be effective agents in attenuating the progression of term labor and preterm labor in human studies [27]. NSAIDs that are currently in clinical use to prevent preterm labor, such as indomethacin, inhibit both COX-1 and 2.

The development of COX-2 specific inhibitors opened new therapeutic options to obstetricians and gynecologists as these drugs have been hailed as better-tolerated alternatives to conventional NSAIDs. COX-2 inhibitors significantly relax the human myometrium. Nonetheless their use is not very popular today due to association between COX-2 inhibitors and cardiovascular (CV) and thromboembolic events. A review of 13 randomized trials was done to assess the effects of NSAIDs as a tocolytic agent on maternal, fetal and neonatal outcomes [28]. NSAIDs were compared with placebo, other tocolytics and no intervention. Ten trials used the non-selective COX inhibitor, indomethacin. Indomethacin use resulted in a reduction in birth before 37 weeks’ gestation, (relative risk (RR) 0.21; one trial, 36 women), an increase in gestational age (weighted mean difference (WMD) 3.53 weeks) and birthweight (WMD 716.34 gm; two trials, 67 women). Two additional trials compared the use of non- selective COX inhibitors *versus* COX-2 inhibitor (rofecoxib and nimesulide) (two trials, 54 women) did not demonstrate any differences in maternal or neonatal outcomes. Compared to any other tocolytic, COX inhibitors were again associated with a decrease in the proportion of women delivering at less than 37 weeks' gestation (RR 0.53), and a trend to a reduction in the proportion of women delivering less than 48 hours from initiation of treatment.

A concern about possible effects of NSAIDs on the fetal ductus arteriosus, antenatal constriction and closure leading to postnatal persistent patent ductus was also addressed in the review mentioned above [28]. In this review, a total of 403 women received short-term NSAIDs tocolysis (up to 48 hours), and there was only one case of antenatal closure of the ductus arteriosus. There was no increase in the incidence of patent ductus arteriosus postnatally. This shows that for a short term use of NSAIDs, the effect on ductus arteriosus may have been overstated. 

Another meta- analysis of fifteen retrospective studies and six case controlled studies found a statistically significant association between periventriclar leukomalacia and administration of antenatal indomethacin in premature infants [29]. In addition, the same study showed a statistically significant association between necrotizing enterocolitis and administration of antenatal indomethacin within 72 hours prior to delivery in premature infants.

Necrotizing enteroclitis with administration of antenatal indomethacin in premature infants. This adverse effect was attributed to the influence of the indomethacin on cerebral and mesenteric blood flow [29]. 

## 8. Conclusions

In this article we have reviewed the specific implications of NSAIDs in obstetrics, gynecology and infertility. These medications were found to be effective not only in pain management, but also had additional beneficial influence such as decreasing menstrual bleeding, preventing premature ovulation in natural IVF cycles and serving as tocolytics in preterm labor. The newer COX-2 specific inhibitors may have a role in the treatment of primary dysmenorrhea, and may be effective in postoperative pain as well as pain associated with endometriosis.
